# Evaluation of the Anti-inflammatory Effects of Ticagrelor and Prasugrel as Add-On Therapy to Aspirin in Diabetic Patients Post-percutaneous Coronary Intervention

**DOI:** 10.7759/cureus.96548

**Published:** 2025-11-11

**Authors:** Ankita Panigrahy, Padmaja Mekala, Usharani Pingali, Bharathilakshmi VS

**Affiliations:** 1 Department of Clinical Pharmacology and Therapeutics, Nizam's Institute of Medical Sciences, Hyderabad, IND; 2 Department of Cardiology, Nizam's Institute of Medical Sciences, Hyderabad, IND

**Keywords:** acute coronary syndrome, anti-inflammatory, diabetes mellitus, prasugrel, ticagrelor

## Abstract

Background

Diabetes mellitus increases the incidence and severity of acute coronary syndromes (ACS). Dual antiplatelet therapy, combining aspirin and P2Y12 (purinergic 2Y type 12) receptor antagonists, is the cornerstone of therapy post-percutaneous coronary intervention (PCI) in diabetic patients. Beyond their antithrombotic effects, P2Y12 inhibitors may exert anti-inflammatory effects by modulating leukocyte-platelet interactions. Hence, this study aimed to investigate the anti-inflammatory effects of adding either ticagrelor or prasugrel to aspirin in diabetic patients with ACS undergoing PCI, by estimating high-sensitivity C-reactive protein (hs-CRP), interleukin-6 (IL-6), and tumour necrosis factor-alpha (TNF-α) levels.

Methods

A prospective, observational, comparative study was conducted to estimate the changes in hs-CRP, IL-6, and TNF-α levels in the serum over 12 weeks of adding either ticagrelor (Group 1) or prasugrel (Group 2) to aspirin in diabetic patients with ACS undergoing PCI.

Results

Forty-six patients, with a mean age of 56 ± 9 years, were included in the study. A significant reduction in the number of patients with hs-CRP levels >3 mg/L at 12 weeks, compared to baseline, was seen in both study groups. Additionally, there was a statistically significant reduction in IL-6 and TNF-α levels, within as well as between the study Group 1 (ticagrelor) and Group 2 (prasugrel), at the end of 12 weeks compared to baseline. No major adverse event was reported in the study.

Conclusion

Both ticagrelor and prasugrel showed anti-inflammatory effects, with ticagrelor showing statistically significant reductions in inflammatory markers, compared to prasugrel, in our study population.

## Introduction

Diabetes mellitus is a well-established risk factor for coronary artery disease, and it significantly increases the incidence and severity of acute coronary syndrome (ACS). Diabetic patients exhibit more extensive, diffuse, and complex coronary atherosclerosis, contributing to heightened cardiovascular morbidity and mortality. Coronary artery disease accounts for up to 75% of mortality in diabetic patients [1].

Reperfusion strategies, particularly percutaneous coronary intervention (PCI), represent the cornerstone of ACS management in diabetic patients. Dual antiplatelet therapy, combining aspirin and P2Y12 (purinergic 2Y type 12) receptor antagonists (clopidogrel, prasugrel, or ticagrelor), is recommended by the 2023 European Society of Cardiology guidelines to optimize post-PCI outcomes [2].

Beyond their antithrombotic effects, P2Y12 inhibitors may exert anti-inflammatory effects by modulating leukocyte-platelet interactions and reducing inflammatory mediators. Inflammation plays a crucial role in all stages of atherothrombosis, with cytokines such as interleukin-6 (IL-6) and tumour necrosis factor-alpha (TNF-α) implicated in plaque destabilization and ACS pathophysiology [3]. Additionally, prior studies have shown that combinations of biomarkers, such as high-sensitivity C-reactive protein (hs-CRP) with GRACE scores (Global Registry for Acute Coronary Events), enhanced risk discrimination in patients with ACS [4]. Yet, the role of hs-CRP as a marker for ACS has not yet been proved.

Cytokines, such as IL-6, have prognostic value in patients with ACS and have a strong association with disease severity. IL-6 levels are closely related to the concentration of hs-CRP, indicating that IL-6 may be involved in the early acute-phase reaction of ACS. Yang et al. have shown that serum hs-CRP and IL-6 levels can be used to determine plaque stability, which is closely related to ACS [5].

Clopidogrel, the most extensively studied P2Y12 inhibitor, has shown anti-inflammatory effects by inhibiting adenosine diphosphate (ADP)-induced P-selectin expression, platelet-leukocyte aggregate formation, and soluble CD40 ligand release [6]. The newer P2Y12 inhibitors, ticagrelor and prasugrel, have the added benefits of increased potency and lower chances for drug-drug interactions compared to clopidogrel.

Ticagrelor, a reversible and direct P2Y12 antagonist, possesses additional pleiotropic properties, notably inhibition of equilibrative nucleoside transporter-1 (ENT-1), leading to increased extracellular adenosine levels. This mechanism is associated with enhanced coronary vasodilation, attenuation of inflammatory cytokines (e.g., IL-6 and TNF-α), and improved endothelial function [7]. Prasugrel, an irreversible prodrug, also demonstrates potential anti-inflammatory effects, including inhibition of platelet-monocyte interactions and cytokine release, although the data remain limited [8].

Studies undertaken recently have shown that these pleiotropic effects may be mediated by adenosine-derived intracellular effects and other pathways, thus regulating vascular tone, inflammation, and endothelial function [9]. In diabetic patients, prasugrel has proven to be beneficial, as per the findings of the TIMI-38 (Thrombolysis in Myocardial Infarction-38) trial [10]. Additionally, ticagrelor has shown marked inhibition of ADP-induced platelet reactivity, in comparison with prasugrel, in patients with ACS, as shown in the OPTIMUS trial [11].

Many clinical trials have tested the anti-inflammatory effects of clopidogrel. Yet, studies with head-to-head comparison of the anti-inflammatory effects of ticagrelor versus prasugrel (as add-on therapy to aspirin) are lacking in the Indian population with diabetes mellitus undergoing PCI. This is significant, as India has a higher prevalence of diabetes mellitus, which is a major risk factor for cardiovascular disease.

Hence, this prospective, observational, comparative study was designed with the primary objective of evaluating the effects of ticagrelor and prasugrel on inflammatory biomarkers (hs-CRP, IL-6, and TNF-α) in diabetic patients with ACS undergoing PCI.

## Materials and methods

The study protocol was approved by the Institutional Ethics Committee (IEC), and the study was conducted in accordance with the principles of the Declaration of Helsinki and Good Clinical Practice Guidelines. Before the enrollment of the study participants, a detailed explanation of the study was given, and they provided written informed consent.

Methodology

This study was conducted in the Department of Clinical Pharmacology and Therapeutics, in collaboration with the Department of Cardiology of Nizam's Institute of Medical Sciences, Hyderabad, India. Ethics Committee approval was obtained from the Institutional Ethics Committee (approval no. 66th ESGS/1500/2023), and the study was registered with the Clinical Trials Registry of India (CTRI/2023/06/054193). It was a prospective, observational, comparative study of 12 months’ duration (August 2023 to July 2024). The study population consisted of diabetic patients with coronary artery disease, fulfilling the following inclusion and exclusion criteria.

The inclusion criteria, such as patients aged 35-70 years of either sex, with diabetes mellitus, and with either stable angina, unstable angina, non-ST-elevation myocardial infarction (NSTEMI), or STEMI, planned for PCI, and with TIMI flow grade 3, were included in the study. The study participants were on stable background medication for at least three months prior to the start of the study.

The exclusion criteria, such as patients with uncontrolled diabetes, posted for coronary bypass surgery, with a history of stroke, transient ischemic attack, or intracranial bleeding at any time, previous cardiac intervention, requiring chronic anticoagulation therapy, with cardiogenic shock or hypovolemia, left ventricular ejection fraction <30%, chronic kidney disease stage 3 and above, with liver function test values more than twice the upper limit of normal, history of gastrointestinal bleeding within the past six months, or a history of hypersensitivity to study drugs, were excluded.

The patients were screened for eligibility at the screening visit, and eligible participants were enrolled in the study. The choice of antiplatelet treatment was at the discretion of the cardiologist. The study participants received either of the following antiplatelet medications: Group 1 (ticagrelor) - 180 mg loading dose (pre-PCI), followed by 90 mg maintenance dose twice a day, in the morning and at night, after food; Group 2 (prasugrel) - 60 mg loading dose (pre-PCI), followed by 10 mg maintenance dose once daily, in the morning, after food.

All the study participants were taking aspirin 75-100 mg once daily, in the morning, after food. They were then asked to take the study medications as prescribed, after food, with 240 mL of water, for a period of 12 weeks. The study participants were asked to come for review visits at 4 and 12 weeks of treatment.

Inflammatory biomarkers, i.e., hs-CRP, IL-6, and TNF-α, were measured from serum samples at 0 weeks (baseline) and 12 weeks. The collected blood sample was centrifuged at 2,000 rpm for 10 minutes, and serum was stored at -80°C until analysis. Data from other laboratory investigations, such as fasting blood sugar, HbA1C, complete blood count, liver function tests, and renal function tests, were noted from patient records at screening and at the end of 12 weeks. The safety and tolerability of subjects were monitored throughout the study.

The primary outcome measure estimated the changes in levels of hsCRP, IL-6, and TNF-α after 12 weeks of treatment, compared to baseline, within the groups as well as between the groups. hsCRP was measured using the hsCRP fast test kit, intended for in vitro quantitative determination in human serum by immunofluorescence assay. The 95th percentile of the concentration for hsCRP is 3 mg/L, with the lower limit of detection ≤0.5 mg/dL, as per the kit’s specifications. In our study, we documented the values of hsCRP for the patients as either >3 mg/L or <3 mg/L, as values >3 mg/L are shown to be associated with a significant increase in coronary events, according to existing literature and American Heart Association guidelines [12].

IL-6 was measured in serum samples of the patients using a 96-well ELISA kit from Biogenix Inc. Pvt. Ltd., Lucknow, India. The kit had a limit of detection of 3.2 pg/mL, with the range of the standard curve being 7.82-500 pg/mL. Similarly, TNF-α levels in serum were measured using a 96-well ELISA kit from Bioassay Technology Lab, Shanghai, China. This kit had a limit of detection of 1.52 ng/L, with the range of the standard curve being 3-900 ng/mL. Quality controls were used to ensure the reliability of the biomarker measurements.

The secondary outcome measures were the number of adverse drug reactions, stent thrombosis, bleeding episodes, or deaths (if any) in either of the study groups.

Statistical analysis

A total of 46 patients were enrolled in the study. The sample size was calculated based on a study conducted by Jeong et al. (2017) [9]. Forty-two patients were found to be sufficient to detect a mean difference of 0.27 pg/mL, with a standard deviation of 0.31 in IL-6 levels between the groups. Considering a dropout rate of 10%, a total of 46 patients needed to be enrolled for the study. The sample size was calculated assuming 80% power and a probability of type 1 error of 0.05.

Statistical analysis was performed using GraphPad Prism version 9.0.2 (GraphPad Software, San Diego, CA, USA) and Microsoft Excel (Microsoft® Corp., Redmond, WA, USA). All study participants who completed 12 weeks of treatment were considered for efficacy analysis. Data were presented as the mean ± standard deviation for normally distributed data. Comparisons within the group were performed using a paired t-test, while between-group comparisons were performed using an unpaired t-test. All participants who were randomized in the study were considered for safety analysis. Safety data were expressed as numbers.

## Results

**Figure 1:**
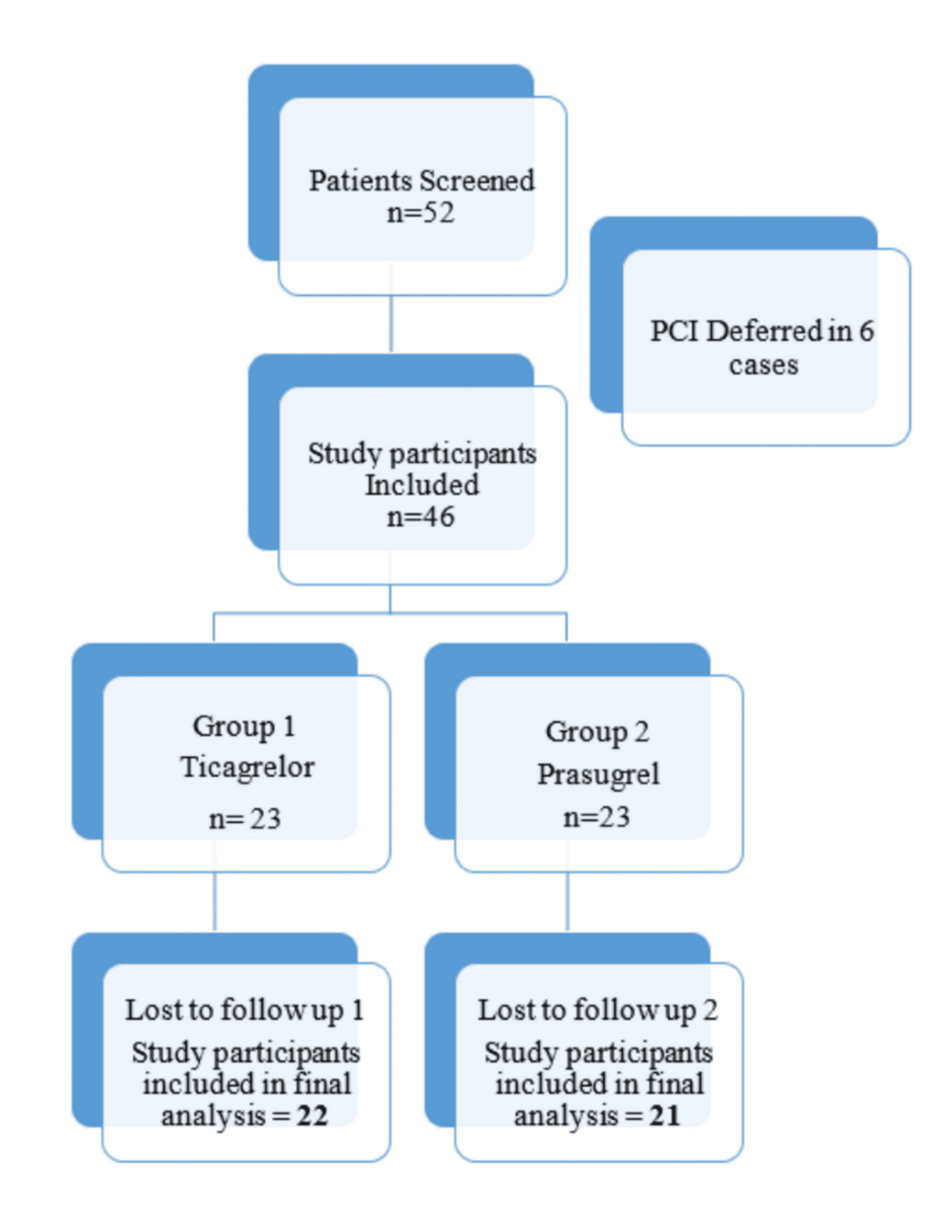
Participant flow diagram

A total of 52 patients were screened, and 46 eligible study participants were enrolled. Six study participants were not included, as the PCI procedure was deferred in them. Out of 46 participants, 43 were included in the final analysis, as three were lost to follow-up. A participant flow diagram is shown in Figure 1.

Of the 43 study participants, 36 were men and 7 were women, with a mean age of 56 ± 9 years. The demographic and baseline characteristics of the study participants are presented in Table 1. Most of the study variables were normally distributed, as determined by the Shapiro-Wilk and Kolmogorov-Smirnov tests. The groups were homogeneous and comparable at baseline (p > 0.05).

**Table 1:** Baseline characteristics Data are expressed as N (%) for discrete variables and as mean ± SD for continuous measures. p < 0.05 was considered statistically significant. M: male; F: female; HbA1c: hemoglobin A1c; hsCRP: high-sensitivity C-reactive protein; IL-6: interleukin-6; TNF-α: tumour necrosis factor-α

Parameter	Group 1 - Ticagrelor (N = 22)	Group 2 - Prasugrel (N = 21)	p-value
Age (years)	55.31 ± 9.55	56.76 ± 9.8	0.60
Gender (M:F)	17:5	19:2	-
BMI (kg/m^2^)	27.96 ± 1.32	27.24 ± 2.70	0.27
Smoker (n)	12 (54.55%)	14 (66.7%)	0.98
Alcoholic (n)	10 (45.45%)	13 (61.9%)	0.36
Hypertensive (n)	15 (68.18%)	16 (76.2%)	0.73
Total cholesterol (mg/dL)	161.45 ± 39.53	152.2 ± 47.04	0.45
Triglyceride (mg/dL)	153.04 ± 49.69	151.71 ± 86.04	0.94
Serum creatinine (mg/dL)	1.15 ± 0.39	0.95 ± 0.19	0.11
HbA1c (%)	7.79 ± 1.76	7.97 ± 1.64	0.81
hsCRP (>3 mg/L)	14 (63.63%)	13 (61.90%)	0.91
IL-6 (pg/mL)	13.07 ± 4.9	11.19 ± 5.24	0.23
TNF-α (pg/mL)	136.9 ± 44.76	117.1 ± 32.04	0.10

Primary outcome measures

**Figure 2:**
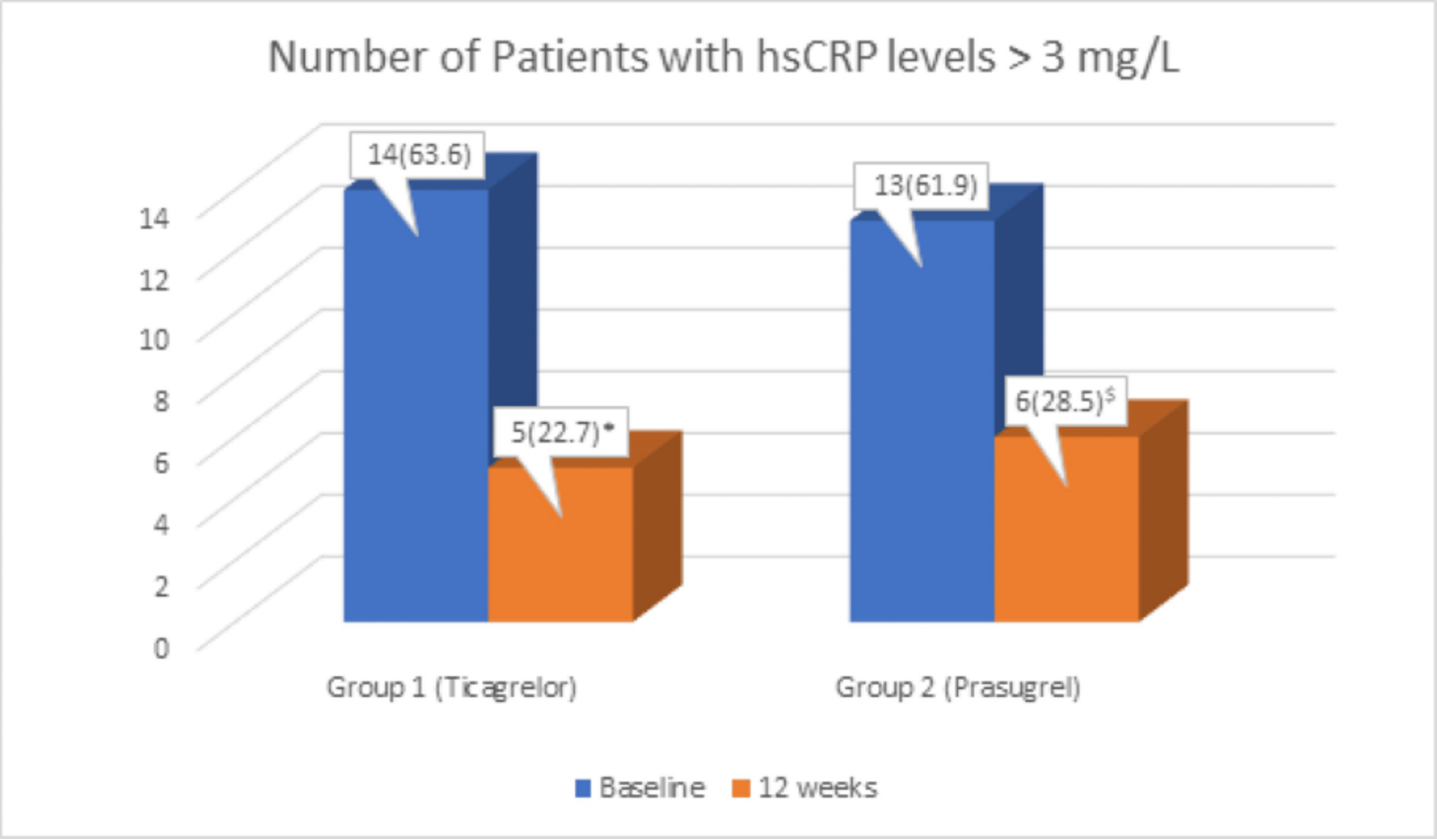
Number of patients with hsCRP >3 mg/L (high risk) at baseline and at 12 weeks Values are presented as N (%). The Chi-square test was used for comparison within the groups (Chi-square value for Group 1 = 7.503, for Group 2 = 4.709). *p = 0.013 compared to baseline for ticagrelor, ^$^p = 0.03 compared to baseline for prasugrel. p < 0.05 was considered statistically significant. hsCRP: high-sensitivity C-reactive protein

Both study groups showed a significant reduction in the number of patients with hsCRP levels >3 mg/L at 12 weeks compared to baseline, as shown in Figure 2. However, there was no significant difference in hsCRP levels between the groups at the end of 12 weeks. 

Both drugs showed a significant reduction in levels of IL-6 and TNF-α at 12 weeks (p < 0.001) compared to baseline. Additionally, there was a significant decrease in levels of IL-6 and TNF-α at 12 weeks in the ticagrelor group (p = 0.031) compared to prasugrel, as shown in Table 2.

**Table 2:** Effect of treatment on IL-6 and TNF-α levels within the study groups Values expressed as mean ± SD. A paired t-test was used for comparison within the groups (t-value of Group 1 for IL-6 = 10.07, TNF-α = 9.335; t-value of Group 2 for IL-6 = 10.68, TNF-α = 9.937). p < 0.05 was statistically significant. IL-6: interleukin-6; TNF-α: tumor necrosis factor-α

Parameter	Group 1 (Ticagrelor)		Within-Group Comparison (12 W-0 W)		Group 2 (Prasugrel)		Within-Group Comparison (12 W-0 W)	
	Baseline	12 weeks	Mean change from baseline	p-value	Baseline	12 weeks	Mean change from baseline	p-value
IL-6 (pg/mL)	13.07 ± 4.9	7.40 ± 3.26	5.66 ± 2.63	<0.001	11.19 ± 5.24	7.06 ± 3.84	4.13 ± 1.774	<0.001
TNF-α (pg/mL)	136.9 ± 44.76	83.47 ± 32.15	53.41 ± 26.84	<0.001	117.1 ± 32.04	81.03 ± 21.02	36.04 ± 16.62	<0.001

**Figure 3:**
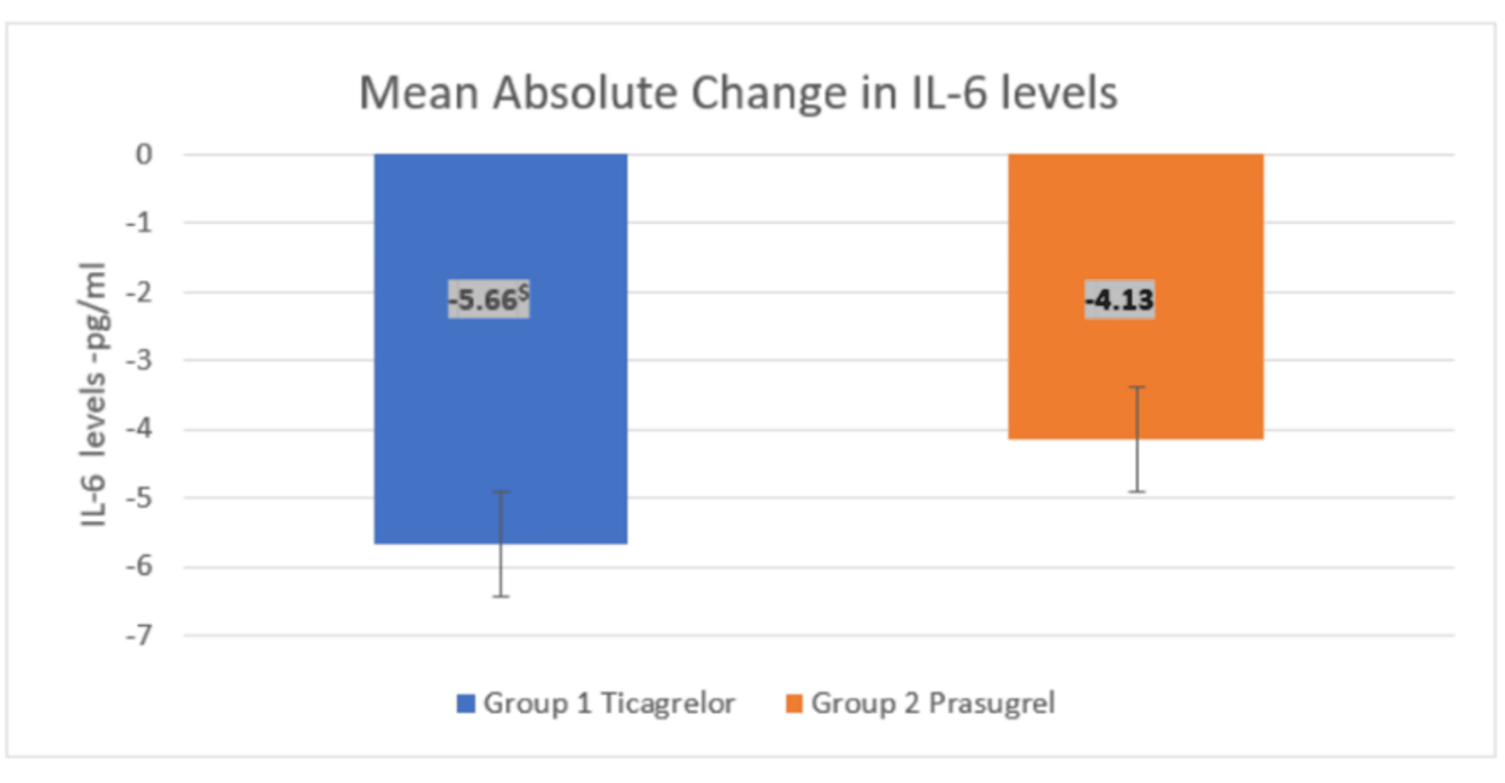
Effect of treatments on mean absolute change in IL-6 levels between the groups at the end of 12 weeks An unpaired t-test was used to compare the absolute change in IL-6 levels between the groups at the end of 12 weeks (t-value = 2.22). ^$^p-value = 0.031 in Group 1 compared to Group 2. p < 0.05 was considered statistically significant. IL-6: interleukin-6

**Figure 4:**
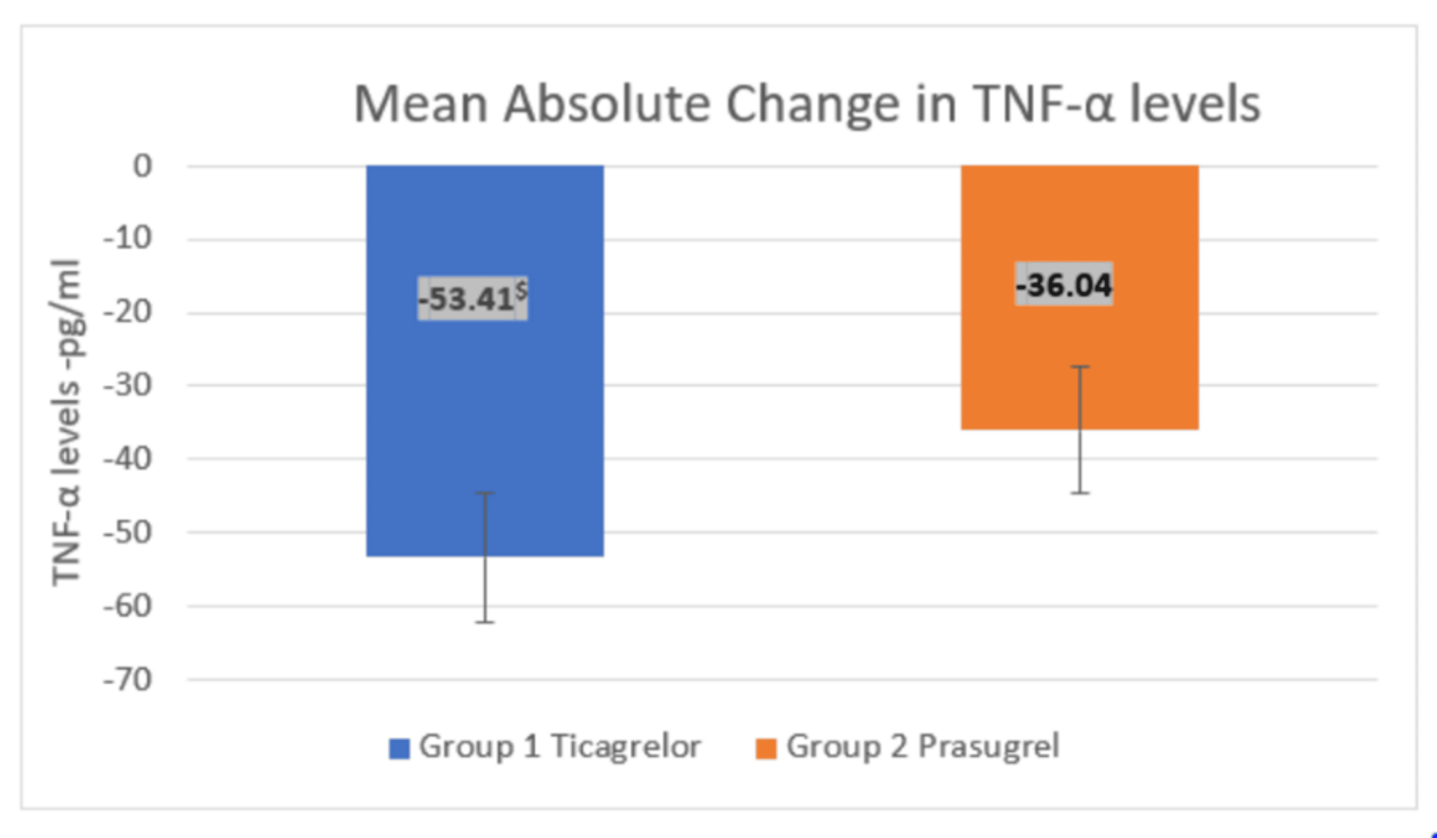
Effect of treatment on mean absolute change in TNF-α levels between the groups at the end of 12 weeks An unpaired t-test was used to compare the absolute change in TNF-α levels between the groups at the end of 12 weeks (t-value = 2.537). ^$^p-value = 0.015 in Group 1 compared to Group 2. p < 0.05 was considered statistically significant. TNF-α: tumor necrosis factor-α

Additionally, there was a significant decrease in the mean absolute change in IL-6 and TNF-α levels with ticagrelor (p = 0.015) compared to prasugrel, from baseline to the end of 12 weeks, as depicted in Figures 3-4.

Secondary outcome measures

A total of eight adverse events were noted in the 43 study participants, as shown in Table 3. The most common adverse event reported was dyspnoea. Dyspnoea is a known adverse effect of ticagrelor, which is mild to moderate in most patients, often occurs in the early stages of treatment, is generally self-limiting, and has no significant effects [13]. All adverse events were managed symptomatically without stopping treatment. No incidence of bleeding, need for restenting, or mortality was observed in any of the study participants.

**Table 3:** Incidence of adverse events Values are expressed as frequencies.

Sl No	Adverse Events	Group 1 - Ticagrelor (n = 22)	Group 2 - Prasugrel (n = 21)
1	Dyspnoea	4	0
2	Gastritis	0	1
3	Nausea	1	1
4	Diarrhoea	1	0
	Total	6	2

## Discussion

Our study is a head-to-head comparison of the anti-inflammatory effects of ticagrelor versus prasugrel as add-on therapy to aspirin in Indian diabetic patients post-PCI. In our study, the mean age of presentation was 56 ± 9 years, which is similar to the studies by Satilmisoglu et al. and Aytekin et al. [14,15]. The gender distribution in our study was 36 males and 7 females. Similar were the findings of Schüpke et al. and Conrotto et al., where 80% to 90% of the patients were males and only up to 20% were females [16,17]. This may be attributed to the lower incidence of ACS in women compared to men, as shown by the GUSTO-IIb trial, where STEMI was significantly less frequent in women than in men (27.2% versus 37.0%) [18].

In the present study, both the study drugs, ticagrelor 90 mg bd and prasugrel 10 mg od, showed a statistically significant reduction in all the inflammatory markers at 12 weeks compared to baseline. We observed a significant reduction in the number of patients with hsCRP levels >3 mg/L at 12 weeks compared to baseline in both study groups. Additionally, there was a statistically significant reduction in IL-6 and TNF-α levels in Group 1 (ticagrelor), as well as in Group 2 (prasugrel), at the end of 12 weeks compared to baseline. The group analysis at the end of 12 weeks showed a statistically significant reduction in levels of IL-6 and TNF-α in Group 1 (ticagrelor) compared to Group 2 (prasugrel).

The anti-inflammatory marker hsCRP, when elevated to >3 mg/L, has been shown to be associated with a significant increase in coronary events compared to a normal value [10]. In our study, it was found that at baseline, more than 60% of the patients had hsCRP >3 mg/dL in both groups. Similar findings have been reported by Rafiqi et al., where more than 50% of ACS patients admitted to the emergency room had hsCRP >2 mg/dL [19]. There was a statistically significant reduction in patients with hsCRP levels >3 mg/L (p < 0.01) at 12 weeks compared to baseline with both ticagrelor and prasugrel, indicating a reduction in cardiovascular risk.

Plasma IL-6 levels are significantly elevated in ACS patients, with the levels sometimes being three times higher than those of healthy controls, as reported in a meta-analysis by Yang et al. [5]. In our study, the diabetic patients with ACS showed a baseline IL-6 value of 13.07 ± 4.9 pg/mL in the ticagrelor group and 11.19 ± 5.24 pg/mL in the prasugrel group. This corroborates a study by Ritschel et al., conducted in 1,028 patients with STEMI, where the mean levels of IL-6 were found to be 18.7 pg/mL [20]. On the other hand, in a study by Rafiqui et al., the baseline IL-6 levels in patients with ACS were lower (median of 2.1 pg/mL, with an IQR of 0.9-5.3). This may be because Rafiqui et al. had only 14% diabetic patients with ACS, which did not include any STEMI patients [19].

In our study, treatment with both ticagrelor and prasugrel showed a statistically significant reduction in levels of IL-6 at 12 weeks compared to baseline. In another study by Lan et al., in patients with myocardial infarction, a statistically significant decrease in IL-6 at the end of three months of treatment with ticagrelor from baseline was reported (from 162.31 ± 33.41 ng/mL to 103.44 ± 12.54 ng/mL). However, their study did not stratify myocardial infarction by diabetic disease state [21]. In contrast to our findings, Jeong et al., in their study involving diabetic patients with NSTEMI, showed significant reductions in IL-6 levels from baseline in the ticagrelor group only, and not with prasugrel [9]. This may be because the study by Jeong et al. involved a crossover design without any washout period in between, which makes attributing the decrease in cytokine levels to either drug challenging.

For the between-groups comparison at the end of treatment, the mean absolute reduction in IL-6 levels was statistically significant in the ticagrelor group compared to prasugrel at the end of 12 weeks (5.66 vs. 4.13, with a p-value of <0.05). This corroborates the findings by Jeong et al., who reported similarly [9]. However, in contrast to our findings, Husted et al. reported that treatment with ticagrelor did not lead to significant changes in IL-6 in patients with NSTEMI [22]. This may be because their study lasted only four weeks, while ours was a 12-week study.

With respect to TNF-α levels, both ticagrelor and prasugrel showed a significant reduction at 12 weeks compared to baseline. Findings by Lan et al. showed a similar statistically significant reduction in TNF-α levels after three months of treatment with ticagrelor [21]. The between-groups comparison in our study showed a statistically significant mean absolute reduction in TNF-α levels in the ticagrelor group compared to prasugrel at the end of 12 weeks. Jeong et al. reported similarly, showing that the mean absolute reduction in TNF-α levels was significantly higher in the ticagrelor group compared to prasugrel (5.62 ± 4.40 pg/mL vs. 0.42 ± 2.64 pg/mL; p < 0.001) [9]. However, the baseline values of TNF-α and the reduction from baseline in their study were lower compared to ours, as they included only NSTEMI patients.

Another effect of ticagrelor is that it may increase adenosine plasma concentration in patients with ACS. At higher concentrations of ticagrelor, adenosine mainly acts on the A2A and A2B adenosine receptors, which subsequently downregulate proinflammatory cytokines, such as IL-6 and TNF-α [23]. Hence, in a high-thrombotic setting, like STEMI or stent thrombosis, choosing the stronger antiplatelet agent may be preferred.

It is also important to note that patients with diabetes mellitus are characterized by increased platelet turnover rates, with newly generated platelets - especially hyperreactive ones - being more frequently released into the systemic circulation. This may explain why the twice-daily administration of ticagrelor is beneficial, as it allows more efficacious platelet inhibition over the 24-hour period compared with prasugrel, which is administered once daily. Additionally, ticagrelor has the benefit of reversibility: it can be stopped three days before surgery, with no anatomical or clinical contraindications.

Evidence from the Indian population, and Asians in general, is especially sparse. A randomized study carried out in Bhopal by Ray et al., in ACS patients receiving either ticagrelor or prasugrel post-PCI, showed that the incidence of bleeding, dyspnoea, or major adverse cardiovascular event (MACE) was not significantly different between the prasugrel and ticagrelor groups [24]. Ndrepepa et al. have shown that the net clinical benefit with either ticagrelor or prasugrel is greater in patients with diabetes mellitus compared with those without diabetes mellitus [25]. On the other hand, a pharmacodynamic study by Erlinge et al. has shown that the effects of prasugrel may be attenuated in patients with diabetes mellitus, as the chronic inflammatory state associated with diabetes mellitus may reduce the metabolic activity of major cytochrome P450 isoforms involved in the biotransformation of prasugrel to its active metabolites [26].

In our study, no patient exhibited any MACE or major bleeding event in either treatment group. Similarly, Fujisaki et al. have reported no significant difference in any safety endpoint post-treatment with ticagrelor and prasugrel in patients with NSTEMI [27].

Strengths and limitations

To our knowledge, our study is the first of its kind in the Indian population in studying the anti-inflammatory effects of ticagrelor and prasugrel on inflammatory markers - hsCRP, IL-6, and TNF-α - in diabetic patients undergoing PCI. The limitation of our study is that the duration was only 12 weeks, and there was a smaller female representation. Randomized controlled trials with a larger sample size and longer duration will further confirm the anti-inflammatory effects of ticagrelor and prasugrel.

## Conclusions

Both ticagrelor and prasugrel have shown a significant reduction in levels of IL-6, TNF-α, and in the number of patients with higher levels of hsCRP. Ticagrelor has shown a significantly greater reduction in IL-6 and TNF-α than prasugrel. Therefore, from this study, it is evident that both ticagrelor and prasugrel have protective effects by virtue of their anti-inflammatory properties, in addition to their antiplatelet actions. Hence, both drugs may be recommended as potential anti-inflammatory agents in diabetic patients with ACS undergoing PCI, with ticagrelor showing a statistically significant reduction in inflammatory markers compared to prasugrel in our study population. 

## References

[1] Babes EE, Bustea C, Behl T (2022). Acute coronary syndromes in diabetic patients, outcome, revascularization, and antithrombotic therapy. Biomed Pharmacother.

[2] Byrne RA, Rossello X, Coughlan JJ (2023). 2023 ESC guidelines for the management of acute coronary syndromes. Eur Heart J.

[3] Thomas MR, Storey RF (2015). Effect of P2Y12 inhibitors on inflammation and immunity. Thromb Haemost.

[4] Klingenberg R, Aghlmandi S, Räber L (2018). Improved risk stratification of patients with acute coronary syndromes using a combination of hsTnT, NT-proBNP and hsCRP with the GRACE score. Eur Heart J Acute Cardiovasc Care.

[5] Yang C, Deng Z, Li J, Ren Z, Liu F (2021). Meta-analysis of the relationship between interleukin-6 levels and the prognosis and severity of acute coronary syndrome. Clinics (Sao Paulo).

[6] Grzesk G, Kozinski M, Navarese EP (2012). Ticagrelor, but not clopidogrel and prasugrel, prevents ADP-induced vascular smooth muscle cell contraction: a placebo-controlled study in rats. Thromb Res.

[7] Alsharif KF, Thomas MR, Judge HM (2015). Ticagrelor potentiates adenosine-induced stimulation of neutrophil chemotaxis and phagocytosis. Vascul Pharmacol.

[8] Totani L, Dell'Elba G, Martelli N, Di Santo A, Piccoli A, Amore C, Evangelista V (2012). Prasugrel inhibits platelet-leukocyte interaction and reduces inflammatory markers in a model of endotoxic shock in the mouse. Thromb Haemost.

[9] Jeong HS, Hong SJ, Cho SA (2017). Comparison of ticagrelor versus prasugrel for inflammation, vascular function, and circulating endothelial progenitor cells in diabetic patients with non-ST-segment elevation acute coronary syndrome requiring coronary stenting: a prospective, randomized, crossover trial. JACC Cardiovasc Interv.

[10] Wiviott SD, Braunwald E, Angiolillo DJ (2008). Greater clinical benefit of more intensive oral antiplatelet therapy with prasugrel in patients with diabetes mellitus in the trial to assess improvement in therapeutic outcomes by optimizing platelet inhibition with prasugrel-thrombolysis in myocardial infarction 38. Circulation.

[11] Franchi F, Rollini F, Aggarwal N (2016). Pharmacodynamic comparison of prasugrel versus ticagrelor in patients with type 2 diabetes mellitus and coronary artery disease: the OPTIMUS (Optimizing Antiplatelet Therapy in Diabetes Mellitus)-4 study. Circulation.

[12] Pearson TA, Mensah GA, Alexander RW (2003). Markers of inflammation and cardiovascular disease: application to clinical and public health practice: a statement for healthcare professionals from the Centers for Disease Control and Prevention and the American Heart Association. Circulation.

[13] Wei P, Wang X, Fu Q, Cao B (2024). Progress in the clinical effects and adverse reactions of ticagrelor. Thromb J.

[14] Satilmisoglu MH, Gul M, Ozyilmaz S, Cizgici AY (2021). Real-life data for major adverse cardiac events in patients with ST-elevation myocardial infarction prasugrel versus ticagrelor. J Physiol Pharmacol.

[15] Aytekin A, Ndrepepa G, Neumann FJ (2020). Ticagrelor or prasugrel in patients with ST-segment-elevation myocardial infarction undergoing primary percutaneous coronary intervention. Circulation.

[16] Schüpke S, Neumann FJ, Menichelli M (2019). Ticagrelor or prasugrel in patients with acute coronary syndromes. N Engl J Med.

[17] Conrotto F, Bertaina M, Raposeiras-Roubin S (2019). Prasugrel or ticagrelor in patients with acute coronary syndrome and diabetes: a propensity matched substudy of RENAMI. Eur Heart J Acute Cardiovasc Care.

[18] Pagidipati NJ, Peterson ED (2016). Acute coronary syndromes in women and men. Nat Rev Cardiol.

[19] Rafiqi K, Hoeks CB, Løfgren B, Mortensen MB, Bruun JM (2023). Diagnostic impact of Hs-CRP and IL-6 for acute coronary syndrome in patients admitted to the ED with chest pain: added value to the HEART score?. Open Access Emerg Med.

[20] Ritschel VN, Seljeflot I, Arnesen H, Halvorsen S, Weiss T, Eritsland J, Andersen GØ (2014). IL-6 signalling in patients with acute ST-elevation myocardial infarction. Results Immunol.

[21] Lan T, Lv L, Gao Q (2022). Effect of ticagrelor combined with acupuncture on myocardial infarction and its effect on levels of serum myocardial enzymes, cytokines and T lymphocytes. Indian J Pharm Sci.

[22] Husted S, Storey RF, Harrington RA, Emanuelsson H, Cannon CP (2010). Changes in inflammatory biomarkers in patients treated with ticagrelor or clopidogrel. Clin Cardiol.

[23] Vilahur G, Gutiérrez M, Casani L (2016). Protective effects of ticagrelor on myocardial injury after infarction. Circulation.

[24] Ray A, Najmi A, Khandelwal G, Jhaj R, Sadasivam B (2024). Comparative effectiveness and safety of prasugrel and ticagrelor in patients of acute coronary syndrome undergoing percutaneous transluminal coronary angioplasty: a propensity score-matched analysis. Indian Heart J.

[25] Ndrepepa G, Kastrati A, Menichelli M (2020). Ticagrelor or prasugrel in patients with acute coronary syndromes and diabetes mellitus. JACC Cardiovasc Interv.

[26] Erlinge D, Varenhorst C, Braun OO (2008). Patients with poor responsiveness to thienopyridine treatment or with diabetes have lower levels of circulating active metabolite, but their platelets respond normally to active metabolite added ex vivo. J Am Coll Cardiol.

[27] Fujisaki T, Kuno T, Briasoulis A, Misumida N, Takagi H, Latib A (2023). P2Y12 inhibitors for non-ST-segment elevation acute coronary syndrome: a systematic review and network meta-analysis. Tex Heart Inst J.

